# Chemical Composition, Physicochemical Characteristics, and Nutritional Value of* Lannea kerstingii* Seeds and Seed Oil

**DOI:** 10.1155/2017/2840718

**Published:** 2017-01-31

**Authors:** Judicaël Thomas Ouilly, Patrice Bazongo, Adjima Bougma, Nèbpawindé Kaboré, Anne Mette Lykke, Amadé Ouédraogo, Imaël Henri Nestor Bassolé

**Affiliations:** ^1^Life and Earth Sciences Training and Research Unit, University Ouaga I Professor Joseph KI-ZERBO, 03 BP 7021, Ouagadougou, Burkina Faso; ^2^Department of Bioscience, Aarhus University, Vejlsøvej 25, 8600 Silkeborg, Denmark

## Abstract

The chemical composition, main physicochemical properties, and nutritional value of seed flour and seed oil of* Lannea kerstingii* were studied. The results indicated that seeds contained 3.61% moisture, 57.85% fat, 26.39% protein, 10.07% carbohydrates, and 2.08% ash. Potassium was the predominant mineral, followed by magnesium and calcium. The essential amino acids were at higher levels than the estimated amino acid requirements of FAO/WHO/UNU except for lysine. Fatty acid composition showed that oleic acid was the major fatty acid, followed by palmitic, linoleic, and stearic acids. Physicochemical properties of the seed oil were melting point, 19.67°C; refractive index (25°C), 1.47; iodine value, 60.72/100 g of oil; peroxide value, 0.99 meq. O_2_/kg of oil; *p*-anisidine value, 0.08; total oxidation (TOTOX) value, 2.06; oxidative stability index (120°C), 52.53 h; free fatty acids, 0.39%; acid value, 0.64 mg of KOH/g of oil; saponification value, 189.73. Total amount of tocopherols, carotenoids, and sterols was 578.60, 4.60, and 929.50 mg/kg of oil, respectively. *γ*-Tocopherol (82%), lutein (80%), and *β*-sitosterol (93%) were the most abundant forms of tocopherols, carotenoids, and sterols, respectively. Seeds of* L. kerstingii* constitute an alternative source of stable vegetable oil and protein for nutritional and industrial applications.

## 1. Introduction

In June 2013, the United Nations projected that the world population will reach 9.6 billion by 2050 from the current 7.4 billion [1]. The world population growth increases the food demand. It is estimated that oil crops must increase by 133 million tonnes to reach 282 million tonnes in order to cover the demand. Four oil crops (oil palm, soybean, rape, and sunflower) account for 83% of the world production [2]. The major oilseed production areas are in the temperate zones. America and Europe together account for more than 60% of the world production of oil seeds, whereas a substantially smaller production (<5%) comes from tropical areas such as Africa, Malaysia, and Indonesia [3]. The most important tropical oil crops include coconut, oil palm, groundnut, and cotton. However, there are many other traditional oil seeds in tropical Africa that are underexploited, as the nutritional and economic values are poorly known. These oils come from numerous botanical families including the Anacardiaceae in West Africa. The Anacardiaceae family comprises about 70 genera and 600 species including oil and protein rich species, for example,* Pistacia vera* L.,* Sclerocarya birrea* (A. Rich.) Hochst., and* Lannea microcarpa* Engl. et K. Krause [4–6].* Lannea kerstingii* Engl. et K. Krause, a close relative to* Lannea microcarpa*, is widely distributed in the sub-Saharan region from Senegal to Cameroon. Oil from* L. kerstingii* seeds is traditionally used in Burkina Faso as food, medicine, and for skin care [7]. However, the proximate, fatty acid, amino acid, vitamin, sterol, and mineral compositions, which reflect nutritional value of seeds, and the physicochemical properties such as melting point, refractive index, iodine value, peroxide value,* p*-anisidine value, acid value, saponification value,* p*-anisidine value, and oxidative stability, which determine the uses and applications of seed oils, have been not yet analyzed for* L. kerstingii* seeds and seed oil. Therefore, this study investigated the chemical composition, the physicochemical properties, and nutritional value of* L. kerstingii* seeds. The work aims to explore potential uses of* L. kerstingii* seeds and seed oil to promote their consumption in local communities and their trade to international markets.

## 2. Material and Methods

### 2.1. Plant Material

Ripe fruits of* L. kerstingii* (30 kg) were collected at Djanga (latitude 10.37 N; longitude 4.47 W) in the Sudanian climatic zone (70–90 rainy days with 900–1200 mm) in southwest Burkina Faso in June 2012 and 2014. A voucher specimen (specimen number 496) was deposited in the herbarium of the University Ouaga I Pr Joseph KI-ZERBO (OUA).

### 2.2. Chemical Analysis of the Seeds

The seed proximate composition was analyzed following the standard official methods of the Association of Official Analytical Chemists (AOAC) [8]: moisture by vacuum oven (method 925.10), crude fat by Soxhlet method (method 960.39), total nitrogen or crude protein by Kjeldahl, using 6.25 as a conversion factor to calculate protein content (method 979.09), and ash by ignition (method 923.03). Carbohydrate content was estimated by difference of mean values, that is, 100 (sum of percentages of moisture, protein, lipid, and ash) [9].

### 2.3. Mineral Content of Seed Flour

To determine the mineral content of seed flour, a 5.0 g sample was incinerated in a furnace at 550°C and the residues were dissolved in 50 mL of 0.5 M HNO_3_ solution. The concentrations of Ca, Na, K, Mg, Zn, and Fe were determined using atomic spectrophotometer (Varian AA240 FS) absorption, following the method of Pinheiro et al. [10]. A calibration curve was prepared using standard metal solutions.

### 2.4. Seed Amino Acid and Seed Oil Fatty Acid Profiles

Official methods of the Association of Official Analytical Chemists were used for the determination of seed flour amino acid (method 982.30) and seed oil fatty acid (methods 996.06, Ce 2-66, 965.49, and 969.33) profiles [11]. Essential amino acid score was calculated with reference to the FAO/WHO/UNU reference amino acid pattern [12] as follows:

(1) $$\begin{array}{c} \text{Amino acid score}=\frac{\text{Test amino acid}}{\text{Reference amino acid}}\times 100. \end{array}$$

### 2.5. Physicochemical Analysis of Seed Oil

Official methods of the American Oil Chemists' Society were used for the determination of melting point (method Cc 1-25), refractive index (method Cc 7-25), iodine value (method Cd 1-25), peroxide value (method Cd 8-53), *p*-anisidine value (Cd 18-90), acid value (method Ca 3a-63), and saponification value (method Cd 3-25) [13]. The total oxidation (TOTOX) value was calculated using determined values for peroxide and *p*-anisidine (2Px + Av) [14]. Stability was measured with a 743 Rancimat instrument (Metrohm, Herisau, Switzerland) using an oil sample of 3 g, warmed to 120°C and an air flow rate of 20 L/h. Stability was expressed as induction time (h).

### 2.6. Tocopherol, Carotenoid, and Sterol Analysis

Carotenoids, tocopherol, and sterols were analyzed at Craft Technologies Inc. (Wilson, NC). Tocopherols were separated and quantified by HPLC according to AOCS method Ce 8-89 [13]. Carotenoids were separated and quantified by reversed-phase HPLC with UV-Vis detection using published methodology by Craft [15]. Sterols were separated and quantified using GC according to AOCS Official Method Ch 6-91 [16].

### 2.7. Statistical Analysis

Results are expressed as the mean and standard deviation of three separate determinations.

## 3. Results and Discussion

### 3.1. Proximate Composition of Seeds

The results of proximate analysis of* L. kerstingii* seeds are shown in Table 1. The moisture content of seeds was 3.61%, which is low and therefore beneficial for prolonging the shelf life of the seeds. The seeds contained significant amounts of crude oil (57.85 g/100 g), crude protein (26.39 g/100 g), and ash (2.08 g/100 g). The ash and crude protein were in the range of those reported for* Arachis hypogaea* L. and* Pentaclethra macrophylla* Benth. [17]. The crude oil content was higher than those of some commercial seed oils, namely, soybean, cotton seeds, and rubber seeds [18].* L. kerstingii* seeds also contained significant amounts of minerals. The most abundant was potassium followed by magnesium, calcium, zinc, iron, and sodium. These results showed that* L. kerstingii* seeds can be regarded as a good source of oil, protein and minerals.

### 3.2. Amino Acid Composition of Seed Flour

Table 2 depicts the amino acid composition of* L. kerstingii* seed flour. The results indicated that the essential amino acids formed 36.48% of the total amino acid content and most were at higher levels than in the requirements recommended by FAO/WHO/UNU [12], with the exception for lysine which appeared as a limiting amino acid. Tryptophan had the highest amino acid score, followed by phenylalanine + tyrosine, histidine, isoleucine, methionine + cysteine, valine, threonine, leucine, and lysine.

Nonessential amino acids represented 53.39% of total amino acid content. The highest levels were recorded for glutamine, followed by arginine, asparagine, glycine, serine, alanine, proline, hydroxylysine, and hydroxyproline. The total amino acid composition of* L. kerstingii* seed flour is far greater than the one of soybean [19].* L. kerstingii* seed flour is rich in both essential and nonessential amino acids. It constitutes a potential source of protein for human food and livestock fodder.

### 3.3. Fatty Acid Composition of Seed Oil

The fatty acid profile of* L. kerstingii* seed oil is shown in Table 3. The oil mainly contained saturated fatty acids (42.14%) and monounsaturated fatty acids (41.09%) and a low amount of polyunsaturated fatty acids (13.05%).

The most abundant fatty acids of* L. kerstingii* seed oil were oleic (38.45%) and palmitic (33.20%) acids followed by linoleic (12.75%) and stearic (7.43%) acids, which together comprised 91.83% of the total fatty acid. The fatty acid composition of* L. kerstingii* seed oil was comparable to that of palm oil and like that one, the* L. kerstingii* seed oil can be regarded as an oleic-palmitic oil [20]. Therefore,* L. kerstingii* seed oil can be considered as an alternative to palm oil in food industries.

### 3.4. Physical Properties of Seed Oil

Physicochemical properties of* L. kerstingii* seed oil are listed in Table 4. The oil was liquid at 19.67°C. The refractive index of oils depends on their molecular weight, fatty acid chain length, degree of unsaturation, and degree of conjugation. The refractive index of* L. kerstingii* seed oil was 1.47, which was similar to the values of* Acacia senegal* (L.) Willd. (1.47) and* Lannea microcarpa* (1.47) and higher than* Phoenix canariensis* Hort. ex Chabaud (1.45) seed oils [5, 21, 22]. The refractive index is positively related to iodine value, which is a measure of the degree of unsaturation of the oils and gives an idea of their oxidative stability.

The iodine value of 60.72 g of I_2_/100 g of oil was in the range of that of* Moringa oleifera* Lam. oil (65.90 g of I_2_/100 g) and lower than those of olive, cotton, groundnut, and sunflower oils, which ranged from 86 to 145 g of I_2_/100 g of oil [21]. The relatively low iodine value implies low nutritional value, but high oxidative stability. The oxidative susceptibility of* L. kerstingii* seed oil was assessed by the determination of peroxide,* p*-anisidine values, and the oxidative stability index.

The peroxide value of the* L. kerstingii* seed oil was 0.99 meq. O_2_/kg of oil, which is less than 10 meq. O_2_/kg of oil, allowed for crude oils by Codex Alimentarius Committee [22].

The* p*-anisidine value of the oil was 0.08 and lower than that of 0.30 reported for* Salvia hispanica* L. seed oil [23]. The total oxidation (TOTOX) value of 2.06 was lower than those of vegetable oils reported in the literature and indicates high primary and secondary oxidative stability [24]. The oxidative stability of* L. kerstingii* seed oil was 52.53 h at 120°C. This value was higher than those of palm kernel oil (26.80 h) and refined-bleached-deodorized palm olein (25.50 h) measured at 110°C [25]. The low level of polyunsaturated fatty acids provides the oil with high oxidative stability [26]. The double bounds in polyunsaturated are more reactive than a double bound in a monounsaturated chain [27]. Consequently, the high level of monounsaturated fatty acids and the high proportion of saturated fatty acids in* L. kerstingii* seed oil are factors that positively contribute to the oil oxidative stability.

The concentration of free fatty acids and the acid value of the* L. kerstingii* seed oil were 0.39% and 0.64 mg of KOH/g of oil, respectively. These low values were a result of lower hydrolysis of triglycerides and signified that the oil could have a long shelf life, which allows it to be consumed as virgin edible oil.

*L. kerstingii* seed oil had a saponification value of 189.73. This value is due to high content of medium chain fatty acids (i.e., C16 and C18).

### 3.5. Vitamins and Sterols

Vitamin E includes four isomers (*α*, *β*, *δ*, and *γ*) of tocopherol and four isomers (*α*, *β*, *δ*, and *γ*) of tocotrienol. The contents of total and individual tocopherols and tocotrienols of* L. kerstingii* seed oil are presented in Table 5. The results revealed the presence of the three tocopherols (*α*, *δ*, and *γ*). Β-Tocopherol and tocotrienols were not detected in the oil. Total amount of tocopherols was 578.60 mg/kg, which was similar to that reported for* L. microcarpa* and higher than that recorded in grapeseed oil (140.60 mg/kg), peanut oil (398.60 mg/kg), and olive oil (216.80 mg/kg) [28]. *γ*-Tocopherol was the most abundant, with a value of 82% of the total tocopherol content, followed by *α*-tocopherol (12%) and *δ*-tocopherol (6%). The antioxidant activity of tocopherols decreased in the order of *γ* > *δ* > *α* > *β* [29]. The significant quantity of *γ*-tocopherols found in* L. kerstingii* seed oil could contribute to its high oxidative stability.

Carotenoids together with tocopherols are involved in the oxidative stability of the oil and have a protective role against cancer and cardiovascular diseases [30]. More than 700 carotenoids have been identified in 89 plant foods and in the human body, but the overwhelming majority (ca. 90%) in the human diet is represented by *β*-carotene, *α*-carotene, lycopene, lutein, cryptoxanthin, and zeaxanthin [31]. The carotenoid content of* L. kerstingii* seed oil was 4.62 mg/kg of oil (Table 5). This value was similar to that reported for* Capparis spinosa* L. (4.57 mg/kg of oil) and lower than those of* Phoenix canariensis* seed oil (55.10 mg/kg of oil) and* Rubus idaeus* L. seed oil (230.00 mg/kg of oil) [14, 29]. The forms of carotenoids found in* L. kerstingii* seed oil were lutein and *β*-carotene. Lutein was the most abundant form, accounting for 80% of the total carotenoids.

Sterols constitute the major fraction of the unsaponifiable matter in many oils. More than 40 phytosterols were identified; from them *β*-sitosterol, campesterol, and stigmasterol account for more than 95% of total phytosterols dietary intake [32]. They are of interest due to their antioxidant activity and beneficial impact on human health [33]. The sterol content of* L. kerstingii* seed oil is illustrated in Table 5. The total sterol content was 929.50 mg/kg of oil. *β*-Sitosterol was the major form (93%), followed by campesterol (7%). The presence of high *β*-sitosterol was found to limit TG polymer formation in triolein, refined canola, high oleic sunflower, and flaxseed oils heated at frying temperature [34]. This suggests that* L. kerstingii* seed oil could be used as frying oil.

## 4. Conclusion

The present study on the chemical composition, physicochemical properties, and nutritional value of* Lannea kerstingii* seeds suggests that these seeds could be considered as an alternative source of oil, protein, and micronutrients. The seed flour contains all essential amino acids in higher values than those listed in the FAO/WHO/UNU standard, except for lysine. The seed oil of* L. kerstingii* is stable and similar to the palm oil and has good contents of tocopherol, sterol, and carotenoid. It can be a sustainable alternative to palm oil in food industries.

## Figures and Tables

**Table 1:** Proximate and mineral compositions of *Lannea kerstingii* seeds.

Components (g/100 g)	Values

Moisture	3.61 ± 0.28
Proteins	26.39 ± 0.39
Crude fats	57.85 ± 1.64
Carbohydrates	10.07 ± 2.85
Ash	2.08 ± 0.79

Minerals (mg/kg)	Values

Potassium	674.18 ± 27.18
Magnesium	317.15 ± 10.59
Calcium	78.33 ± 4.52
Zinc	6.34 ± 0.08
Iron	4.46 ± 0.05
Sodium	2.48 ± 0.28

Mean values ± standard deviation for *n* = 3.

**Table 2:** Amino acid content of seed flour from *Lannea kerstingii*.

	Amino acid content (g/100 g of protein)	Requirement for adults^a^	Amino acid score
Essential amino acids			
Tryptophan	1.25 ± 0.01	0.6	2.08
Phenylalanine + tyrosine	7.45 ± 0.23	3.8	1.96
Histidine	2.38 ± 0.06	1.5	1.59
Isoleucine	4.53 ± 0.06	3.0	1.51
Methionine + cysteine	3.06 ± 0.08	2.2	1.39
Valine	5.26 ± 0.04	3.9	1.35
Threonine	2.89 ± 0.09	2.3	1.26
Leucine	6.67 ± 0.16	5.9	1.13
Lysine	2.99 ± 0.10	4.5	0.66
Nonessential amino acids			
Glutamine	18.20 ± 0.44		
Arginine	11.01 ± 0.29		
Asparagine	8.19 ± 0.19		
Glycine	4.33 ± 0.13		
Serine	3.92 ± 0.17		
Alanine	3.68 ± 0.10		
Proline	3.36 ± 0.07		
Hydroxylysine	0.45 ± 0.01		
Hydroxyproline	0.25 ± 0.16		
Total essential amino acids	36.48		
Total nonessential amino acids	53.39		

Mean values ± standard deviation for *n* = 3.

^a^Amino acid requirements for adults over 18 years of age [12].

**Table 3:** Fatty acids content of *Lannea kerstingii* seed oil.

Fatty acids	Content (%)
Myristic (14:0)	0.20 ± 0.00
Palmitic (16:0)	33.20 ± 0.42
Palmitoleic (9c-16:1)	0.12 ± 0.01
Margaric (17:0)	0.17 ± 0.00
Stearic (18:0)	7.43 ± 0.10
Elaidic (9t-18:1)	1.53 ± 0.05
Oleic (9c-18:1)	38.45 ± 0.05
Vaccenic (11c-18:1)	0.79 ± 0.02
Linoleic (18:2n6)	12.75 ± 0.06
Linolenic (18:3n3)	0.30 ± 0.00
Arachidic (20:0)	0.94 ± 0.05
Gondoic (20:1n9)	0.20 ± 0.00
Behenic (22:0)	0.20 ± 0.01
SFA	42.14
MUFA	41.09
PUFA	13.05
Total	96.28

Content is expressed as mean values ± standard deviation for *n* = 3. SFA = saturated fatty acid; MUFA = monounsaturated fatty acid; PUFA = polyunsaturated fatty acid.

**Table 4:** Chemical and physical properties of *Lannea kerstingii* seed oil.

Properties	Values
Saponification value (mg KOH/g of oil)	189.73 ± 5.20
Iodine value (g of iodine/100 g of oil)	60.72 ± 3.56
Acid value (mg of KOH/g of oil)	0.64 ± 0.05
Peroxide value (meq of O_2_/kg of oil)	0.99 ± 0.05
Melting point (°C)	19.67 ± 3.80
*p*-Anisidine value	0.08 ± 0.01
Refractive index (25°C)	1.471 ± 0.000
Oxidative stability at 120°C (h)	52.53 ± 2.23

Values are means ± standard deviation for *n* = 3.

**Table 5:** Carotenoid, tocopherol, and sterol contents of *Lannea kerstingii* seed oil.

Carotenoids	Oil content (mg/kg of seed oil)
*cis*-Lutein	3.68 ± 0.03
*trans*-*β*-Carotene	0.94 ± 0.03
Total carotenoids	4.62 ± 0.00

Tocopherols	Oil content (mg/kg of seed oil)

*α*-Tocopherol	70.05 ± 3.75
*β*-Tocopherol	Nd
*γ*-Tocopherol	473.50 ± 16.70
*δ*-Tocopherol	35.05 ± 1.55
Total	578.60 ± 22.00

Sterols	Oil content (mg/kg of seed oil)

Campesterol	66.00 ± 8.00
*β*-Sitosterol	863.50 ± 28.50
Total sterols	929.50 ± 36.50

The contents are expressed by the mean values ± standard deviation for *n* = 3. Nd: not detected.

## References

[1] United Nations World Population Prospects. http://www.un.org/.

[2] FAO (2012). World Agriculture towards 2030/2050: the 2012 Revision. *ESA Working Paper*.

[3] Sharma M., Gupta S. K., Mondal A. K., Gupta S. K. (2012). Production and trade of major world oil crops. *Technological Innovations in Major World Oil Crops: Breeding*.

[4] Aslan M., Orhan I., Şener B. (2002). Comparison of the seed oils of *Pistacia vera* L. of different origins with respect to fatty acids. *International Journal of Food Science and Technology*.

[5] Bazongo P., Bassolé I. H. N., Nielsen S., Hilou A., Dicko M. H., Shukla V. K. S. (2014). Characteristics, composition and oxidative stability of *Lannea microcarpa* seed and seed oil. *Molecules*.

[6] Ogbobe O. (1992). Physico-chemical composition and characterisation of the seed and seed oil of *Sclerocarya birrea*. *Plant Foods for Human Nutrition*.

[7] Ouédraogo A., Lykke A. M., Lankoandé B., Korbéogo G. (2013). Potentials for promoting oil products identified from traditional knowledge of native trees in Burkina Faso. *Ethnobotany Research and Applications*.

[8] AOAC (1999). *Official Methods of Analysis of the Association of Official Analytical Chemists*.

[9] Barminas J. T., James M. K., Abubakar U. M. (1999). Chemical composition of seeds and oil of *Xylopia aethiopica* grown in Nigeria. *Plant Foods for Human Nutrition*.

[10] Pinheiro C., Baeta J. P., Pereira A. M., Domingues H., Ricardo C. P. (2010). Diversity of seed mineral composition of *Phaseolus vulgaris* L. germplasm. *Journal of Food Composition and Analysis*.

[11] AOAC (2006). *Official Methods of Analysis of the Association of Official Analytical Chemists*.

[12] FAO/WHO/UNU (2007). Energy and protein requirements. *WHO Technical Series*.

[13] AOCS (1990). *Official Methods and Recommended Practices of the American Oil Chemists' Society*.

[14] Oomah B. D., Ladet S., Godfrey D. V., Liang J., Girard B. (2000). Characteristics of raspberry (*Rubus idaeus* L.) seed oil. *Food Chemistry*.

[15] Craft N. E. (2001). Innovative approaches to vitamin A assessment. *Journal of Nutrition*.

[16] AOCS (2003). *Official Methods and Recommended Practices of the American Oil Chemists' Society*.

[17] Onyeike E. N., Acheru G. N. (2002). Chemical composition of selected Nigerian oil seeds and physicochemical properties of the oil extracts. *Food Chemistry*.

[18] Ajiwe V. I. E., Umerie S. C., Okeke C. A., Oburota V. N. (1994). Extraction and utilisation of cassava seed oil. *Bioresource Technology*.

[19] Esteves E. A., Martino H. S. D., Oliveira F. C. E., Bressan J., Costa N. M. B. (2010). Chemical composition of a soybean cultivar lacking lipoxygenases (LOX2 and LOX3). *Food Chemistry*.

[20] Tan C.-H., Ghazali H. M., Kuntom A., Tan C.-P., Ariffin A. A. (2009). Extraction and physicochemical properties of low free fatty acid crude palm oil. *Food Chemistry*.

[21] Nehdi I. A., Sbihi H., Tan C. P., Zarrouk H., Khalil M. I., Al-Resayes S. I. (2012). Characteristics, composition and thermal stability of *Acacia senegal* (L.) Willd. seed oil. *Industrial Crops and Products*.

[22] Firestone D. (1997). *Physical and Chemical Characteristics of Oils, Fats and Waxes*.

[23] Codex Alimentarus (2009). *Codex Standard for Edible Fats and Oils Not Covered by Individual Standards. CODEX STAN 19-1981, Rev. 2-1999*.

[24] Ixtaina V. Y., Nolasco S. M., Tomás M. C. (2012). Oxidative stability of chia (*Salvia hispanica* L.) seed oil: effect of antioxidants and storage conditions. *Journal of the American Oil Chemists' Society*.

[25] Shukla V. K., Perkins E. G. (1998). Rancidity in encapsulated health-food oils. *International News on Fats, Oils and Related Materials*.

[26] Tan C. P., Che Man Y. B., Selamat J., Yusoff M. S. A. (2002). Comparative studies of oxidative stability of edible oils by differential scanning calorimetry and oxidative stability index methods. *Food Chemistry*.

[27] Lovato L., Pelegrini B. L., Rodrigues J., Braz de Oliveira A. J., Piloto Ferreira I. C. (2014). Seed oil of *Sapindus saponaria* L. (Sapindaceae) as potential C16 to C22 fatty acids resource. *Biomass and Bioenergy*.

[28] Xu Y. X., Hanna M. A., Josiah S. J. (2007). Hybrid hazelnut oil characteristics and its potential oleochemical application. *Industrial Crops and Products*.

[29] Tuberoso C. I. G., Kowalczyk A., Sarritzu E., Cabras P. (2007). Determination of antioxidant compounds and antioxidant activity in commercial oilseeds for food use. *Food Chemistry*.

[30] Yoshida Y., Niki E., Noguchi N. (2003). Comparative study on the action of tocopherols and tocotrienols as antioxidant: chemical and physical effects. *Chemistry and Physics of Lipids*.

[31] Gimeno E., Calero E., Castellote A. I., Lamuela-Raventós R. M., De La Torre M. C., López-Sabater M. C. (2000). Simultaneous determination of *α*-tocopherol and *β*-carotene in olive oil by reversed-phase high-performance liquid chromatography. *Journal of Chromatography A*.

[32] Calpe-Berdiel L., Escolà-Gil J. C., Blanco-Vaca F. (2009). New insights into the molecular actions of plant sterols and stanols in cholesterol metabolism. *Atherosclerosis*.

[33] Richardson D. G. (1996). The health benefit of eating hazelnuts: implications for blood lipid profiles, coronary heart disease and cancer risks. *Acta Horticulturae*.

[34] Singh A. (2013). Sitosterol as an antioxidant in frying oils. *Food Chemistry*.

